# Velocity correlated crack front and surface marks in single crystalline silicon

**DOI:** 10.1038/s41467-018-03642-w

**Published:** 2018-04-03

**Authors:** Lv Zhao, Didier Bardel, Anne Maynadier, Daniel Nelias

**Affiliations:** 1 Univ Lyon, INSA-Lyon, CNRS UMR5259, LaMCoS, Lyon, F-69621 France; 2 Present Address: Department of Applied Mechanics, FEMTO-ST Institute, Univ Bourgogne Franche Comté, CNRS/UFC/ENSMM/UTBM, Besançon, F-25000 France

## Abstract

Single crystalline silicon fractures on low-energy cleavage planes such as (111) and (110). The crack propagation cannot accurately be predicted by linear elastic fracture mechanics since it does not account for small scale and inelastic phenomena such as atomic lattice trapping. Here we show that, under pure bending load, (110) cleavage in silicon single crystal rapidly accelerates to 3700 m/s without crack path deviation or crack branching, contrasting previous observations. We highlight that the crack front shape involves strong velocity dependence and presents a curvature jump during very high-speed crack growth. In addition, we observe special marks—a kind of periodic surface undulation—that exclusively arise on the rapid fracture surfaces, and we suggest that they are front wave traces resulting from an intrinsic local velocity fluctuation. This finding gives insight to the wavy nature of the crack front in the absence of material asperity.

## Abstract (web summary)

Single crystal silicon cracking, a problem in solar cell operation, remains difficult to accurately predict. Here, the authors show that a silicon single crystal surprisingly cleaves without crack deviation, and that the crack front is accompanied by special marks due to local velocity changes.

## Introduction

The fracture behavior of silicon has received substantial attention over the last few years in relation to its anisotropic behavior, and also for a very local mechanism called atomic lattice trapping which influences the early stage of crack propagation^1, 2^. The fracture of silicon is also of high interest for solar cell applications, given that the solar cells are subjected to vibrations and impacts during transportation and mounting prior to using, and also to severe thermal cycles, hail impact forces and wind vibration during operation.

Single crystalline silicon breaks up preferentially on low-energy crystallographic planes such as (111) and (110)^3–7^. The brittle characteristic makes the fracture a rapid dynamic process with a propagation velocity comparable to the surface wave speed^8–11^. Slow crack growth ($\ll2000$ m/s) has barely been observed in experiments^6, 8^, indicating the existence of a threshold crack velocity in silicon. The velocity gap between 0 and the threshold is attributed to a localized phase transformation before reaching the critical load^12^. This local atom rearrangement actively blunts the crack tip and necessitates a higher energy flux than the intrinsic material toughness upon the crack initiation. The fracture along the [100] direction on the (110) plane (shortened as (110) [100]) cannot be achieved; a systematical deflection onto a (111) plane has been observed in tensile tests^13^. Pérez and Gumbsch^14^ provided an explanation for this deflection phenomenon by means of quantum mechanical calculations. The atomistic fracture scenario revealed that the bond breaking process along (110) [100] path is discontinuous and can cause a large lattice trapping^15^. Another kind of large plane deflection—from (110) [110] to (111)—has been observed under 3-line bending condition^11, 16^. This deflection involved velocity dependence and occurred when the crack propagated faster than 1700 m/s. The authors explained the deflection as a result of the dynamic toughness evolution, based on a postulated phonon emission mechanism^17, 18^. However, according to the aforementioned mechanism, the dynamic toughness of the (110) crystal plane is markedly different from the experimentally measured one^8^. This difference acts against the generalization of the postulated theory and thus the explanation for the plane-switch scenario. Yet, the discussion on the deflection origin has never incorporated the contact effect, note that the 3-line bending introduces a complex stress distribution in the vicinity of the contact line—which is also the region where the crack path deviation has been observed.

Whereas the global fracture behavior of silicon has been widely investigated, the local fracture behavior is less known and needs further exploration, especially under bending load where the crack front is curved and a local crack velocity gradient is present. Fracture surface marks constitute the only way to reconstruct a high-speed fracture process since they are highly correlated with the crack front^19–22^. The primary surface marks are Wallner lines^23, 24^, which involve the interaction between shear waves and the propagating crack front. Wallner lines emanate essentially from surface defects of the specimen or external ultrasonic sources^25^. Secondary fracture surface marks can be attributed to front wave (FW) traces. FWs are solitary waves that propagate only along the crack front at approximately the Rayleigh wave speed (*C*_R_). Initially, these waves have been numerically predicted and depicted as propagating velocity fluctuations confined to the fracture plane^26, 27^. The first experimental observation of FW traces has been reported by Sharon et al.^28, 29^ when exploring the fracture surface of brittle soda-lime glass plates in tensile tests. However, this dissertation led to severe polemics in the fracture community. Bonamy and Ravi-Chandar^24^ suggested that the highlighted marks in refs. ^28, 29^ could completely be interpreted as Wallner lines. Indeed, when a solid fractures under tensile loading, FW traces and Wallner lines would have very similar shapes because of the straight crack front and the close velocities of the two source waves^28^. In the experiments of Sharon and co-workers, due to the material asperity introduced at middle thickness of the sample, FWs and shear waves would emanate simultaneously from the same spot and then overlap one another. The generated surface marks therefore cannot be isolated for meticulous identification, albeit their decay characteristics are different^30^. Hence, exploration and discussion on FW observation remain open.

This work focuses on both global and local fracture behaviors of (110) [110] cleavage within a silicon single crystal, under pure bending. Overall crack path and fracture surface marks are addressed in combination with crack steady-state velocity. We show that (110) [110] cleavage neither involves cleavage plane deflection nor crack branching, even in an extremely high-speed fracture process (3700 m/s). This finding is contradictory with the aforementioned observations in 3-line bending tests. Thanks to meticulous fractographic analysis, it is for the first time highlighted that the crack front shape is strongly dependent on the crack velocity and involves a curvature jump when propagating faster than 2800 m/s. Jointly with the curvature jump, special surface marks (SSMs) have been observed on the fracture surface. These marks are periodic undulations and involve significantly different features from the Wallner lines (also present), and we propose that they are FW traces resulting from a fracture toughness induced local velocity fluctuation.

## Results

### Stability of (110) [110] cleavage

**Fig. 1:**
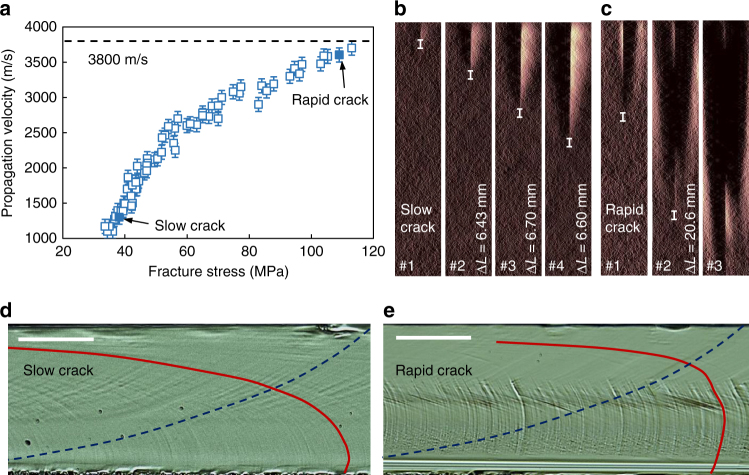
(110) cleavage of silicon single crystal with different crack propagation velocities. **a** Synthesis of the crack speed measurements. **b**, **c** Successive image subtractions from the one prior to cracking, for a slow and a rapid crack, identified by the two solid squares in **a**. The white bar reveals the uncertainty for the crack-tip position (five pixels). **d**, **e** Relatively smooth crack surface for the crack that propagated at a slow speed and the rough one for the fast propagation, respectively. The red solid lines and blue dashed ones highlight the Wallner lines and atomic debonding paths, respectively. The cracks propagate from the left to the right. Scale bars: 100 μm

**Fig. 2:**
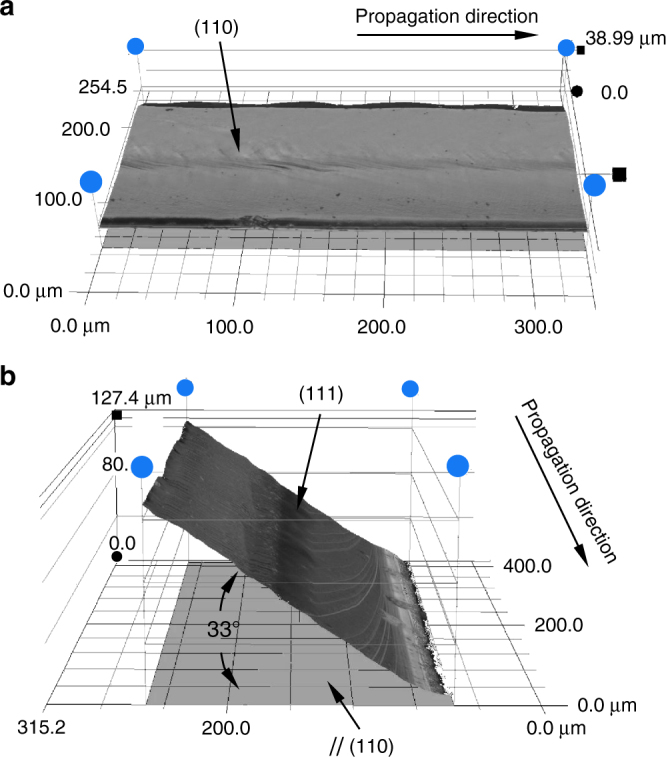
Crack surface topography for the fast crack that propagated at 3600 m/s. **a** Primary crack that initiates from the pre-crack. **b** Secondary crack after deviating underneath the punch roller

Thanks to various pre-crack sizes (from about 200 to 1000 μm) and a substantial amount of experimental data (90 tests), a large range of crack steady-state velocities have been obtained, from about 1050 m/s (0.23 *C*_R_) to 3700 m/s (0.83 *C*_R_), as shown in Fig. 1a. The steady-state velocities are obtained thanks to high-speed imaging technique as follows: the crack-tip position is highlighted by performing subtractions between sequential images after cracking and the last image before cracking. The subtraction results are then subjected to contrast reinforcement as well as denoising processing (Matlab Wavelet algorithm) to have an accurate crack length determination, as shown in Fig. 1b, c for a representative slow crack and a rapid crack, respectively. The crack velocity is calculated by analyzing the results image after image, based on a time step of 5.6 μs. The velocity uncertainty is about 100 m/s while the uncertainty on the crack-tip location is five pixels (equivalent to 0.6 mm). Here the representative slow crack corresponds to a velocity of 1200 m/s whereas the rapid one propagates at 3600 m/s. The two cracks involve well-distinguished fracture surface morphologies, as can be noticed in Fig. 1d, e. Results for other cracks are summarized in Supplementary Fig. 1. Both slow and rapid cracks propagate on the (110) plane without noticeable deflection onto the (111) plane (see Fig. 2a for the rapid crack). In fact, no deflection has been observed on any primary crack path (initiated from the pre-crack) in our wide set of experimental data presented in Fig. 1a. The deflection free (110) [110] cleavage up to 3700 m/s in this work contradicts what has been reported previously; however, only observed under 3-line bending condition^11, 16^. One may assume that the deflection revealed in previous works is linked to the complex stress gradient generated by the contact with the punch roller. Conversely, we believe our observations are more reliable, since the cracks undergo a contact-free stress field for 4-line bending. Indeed, the stress field below the contact line may promote lateral shearing, which impacts the crack path. This assumption has been verified in our work: when the primary crack propagated at around 3600 m/s, secondary cracks nucleated underneath the punch rollers. These cracks tended to propagate on a (111) plane, as shown in Fig. 2b. Note that the measured tilt angle is about 33°, close to the theoretical angle between the (110) and (111) planes, i.e., 35.2°. Therefore, we can conclude that the (110) [110] cracking is very stable in perturbation-free regions, and that the deflection phenomenon is a result of contact effect rather than thermal phonon emission. On the other hand, the proposed phonon emission mechanism^17, 18^ should be considered with caution.

### Crack front evolution

**Fig. 3:**
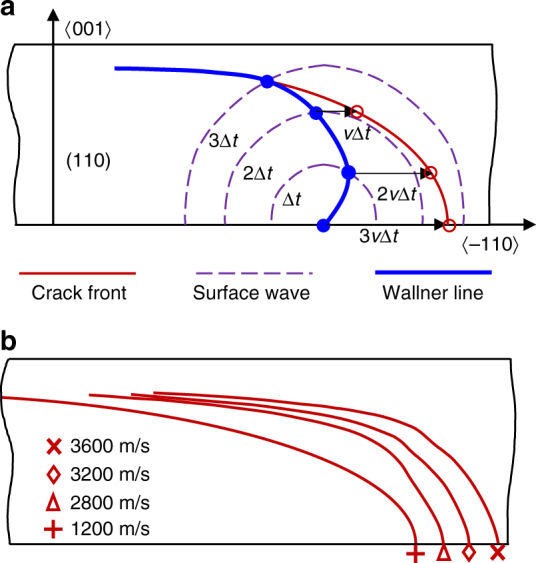
Crack front identification, method and results. **a** Method for recovering the crack front shape from Wallner lines and surface waves. The blue solid line denotes the crack front, the dashed ones stand for the propagating surface waves, and the red one represents the Wallner line. **b** Crack front shapes for a slow crack propagating at 1200 m/s and a rapid one at 3600 m/s, along with cracks propagating at intermediate velocities. Note that the front for rapid cracks presents a curvature jump while that for the slowest one is smooth

In our bending configuration, the crack front is curved due to the stress gradient in the specimen thickness, and the local velocity will decrease from the tensile side to the compression side. In such a case, the knowledge of the crack front shape can help us to assess the local velocities and investigate the local fracture behavior which can only be inferred by postmortem analysis with fracture surface marks. In the smooth region, both (slow crack and rapid crack in Fig. 1b, c) fracture surfaces contain successive-hook like marks initiated from lower surface defects, as shown in Fig. 1d, e. These marks are very typical Wallner lines in bending fracture tests^16, 31^. By observing the Wallner line shape and knowing the Rayleigh wave velocity^32^ as well as the crack propagation speed *v*, the real crack front shape can be addressed in the inverse sense of Wallner line generation. The method is based on the principle such that each point on the Wallner line belongs to the crack front during its propagation. As schematized in Fig. 3a, we choose a fixed time increment Δ*t* and discretize the Wallner line from the lower part to the upper part with a couple of (*n*) surface waves, then we conduct horizontal translations from the last discretized point to the lowest point, with translation distances 0, Δ*t*·*v*, 2Δ*t*·*v*, …, *n*Δ*t*·*v*. This way we will get *n* new points, including the last discretized point on the Wallner line. The crack front shape can be finally obtained by connecting these new points. With this method, the crack fronts for the slow crack (1200 m/s) and the rapid one (3600 m/s) as well as for two additional cracks that propagated at intermediate velocities have been determined and plotted in Fig. 3b. The front for the slowest crack can be approximated with an ellipse of semi-axes *a* = 3*h* and *b* = 0.85*h* (*h* stands for the specimen thickness), which agrees well with the conclusion inferred from a propagation velocity lower than 1700 m/s by Sherman and Be’ery^16^. This previous work did not address the front shape for any higher crack velocity since the fracture experiments involved a deflection from the (110) plane to the (111) plane, where the surface instabilities^33^ concealed the Wallner lines. Nevertheless, thanks to the stable propagation in a contact-free region, crack front shapes for rapid propagation cases (>2000 m/s) are for the first time highlighted in this work. From Fig. 3b, one can notice that the front shape of high-speed crack is complex and cannot be described by a simple basic shape or function, mainly due to the curvature jump at a certain height. A meticulous analysis has permitted to identify the critical speed at which a curvature jump can be observed. This curvature jump onset velocity is about 2700–2800 m/s. Another intriguing observation involves the significant evolution of the crack front shape as a function of the crack propagation velocity under identical loading conditions. This velocity dependence feature is related to our bending configuration and not expected in tensile one. Note that the crack front is directly linked to the atomic debonding process, i.e., local velocity; the front shape evolves as long as the local velocity distribution alters. Under bending condition, the local crack velocity at the tensile side (or lowest point along the crack front) coincides with the global crack velocity, which is also the one measured by real-time diagnostics. Then it decreases and tends toward 0 in the upper region since the crack front tends to an asymptotic horizontal line (see Fig. 3b). For instance, for any two cases with different global velocities, the local velocity distributions are markedly different across the specimen thickness. Consequently, the crack front changes from one case to the other as it accommodates a dissimilar velocity distribution. Thanks to the determined crack front shape and the measured global crack velocity, it is then possible to address the local crack velocity as well as the energy release rate at any locus of the crack front. In practice, the consideration of velocity correlated crack front shape is of tremendous importance in order to ensure high accuracy in such analysis. Moreover, the crack front shape in (111) cleavage should be determined in a dedicated fracture analysis rather than referring that in (110) cleavage at the same global crack velocity, given that the material toughness is quite different between the two crystal planes^5^. However, these two points have not been properly taken into account in the previous studies^16, 33^.

### Secondary special surface marks

**Fig. 4:**
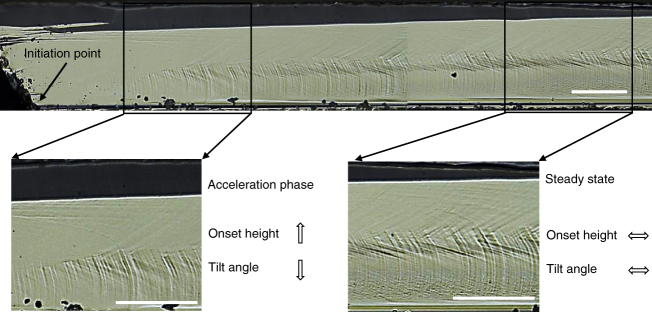
Crack surface morphology nearby the crack initiation spot for the rapid crack (3600 m/s). SSMs appear about 150 μm away from the initiation site and their position moves up in thickness as the crack velocity increases. Scale bars: 100 μm

Particularly, for the rapid crack (3600 m/s), besides the Wallner lines, one may observe some SSMs and eventually some local tiny instabilities. These marks arise just after the crack initiation and cover continuously the whole crack surface. In fact, SSMs have been systematically observed on the fracture surface of specimens when the crack propagation velocity is higher than 2800 m/s, jointly with the presence of a curvature jump along the crack front shape. According to the observations of fracture surfaces in the steady-state phase (see Supplementary Fig. 1), these special marks arise at about 0.34*h* with a tilt angle of 30° for 2800 m/s, while at about 0.58*h* with a tilt angle of 12° for 3700 m/s. Both the onset height and the tilt angle involve a strong dependence on the crack velocity, as summarized in Supplementary Fig. 2. This dependence can be more clearly noticed on the fractography near the crack initiation point for the rapid crack (3600 m/s). As presented in Fig. 4, the SSMs appear 150 μm away from the crack initiation site, their vertical position then moves up from about 0.35*h* to 0.55*h* (at 1000 μm) as the crack propagation velocity increases up to the steady-state regime with a stable position of 0.57*h*.

**Fig. 5:**
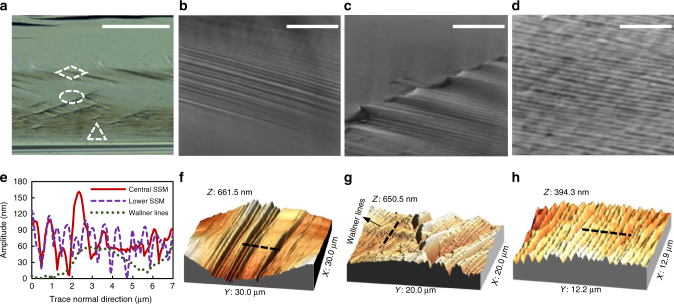
Special surface marks on the (110) plane for a rapid propagation. **a** Digital image showing different marks at different loci. **b**–**d** Local details obtained by SEM which correspond to the framed regions in parallelogram, ellipse, and triangle, respectively. **e** Profile (wavelength and amplitude) of the Wallner lines and the SSMs measured along the dashed lines presented in **f**–**h**. **f**–**h** Topographies of the SSM in the regions corresponding to **b**–**d**. Scale bars: **a** (100 μm), **b** (10 μm), **c** (10 μm), and **d** (5 μm)

In order to address more accurately the geometrical features of the SSMs, we focus our attention on the sample with a crack velocity of 3600 m/s. A representative region is illustrated in Fig. 5a with a digital microscopic image, and then detailed with SEM observations in Fig. 5b–d and AFM topographies in Fig. 5f–h (the SEM images and AFM topographies have up–down correspondence in Fig. 5). According to Fig. 5b, the central SSMs are almost straight and tilted by 12° from the horizontal direction. This angle allows us to rule out the interpretation of SSM based on dislocation. The lower SSMs all present a similar tilt angle, as shown in Fig. 5d. They seem to be extension of the central SSMs. Tiny instabilities eventually arise as presented in Fig. 5c. AFM topographies provide more readable characteristics of the SSMs at the same regions. Figure 5f, h shows that the SSMs are in fact small-amplitude surface undulations rather than micro-plane deflections. Concerning the local instabilities or marked undulations that one may observe in Fig. 5g, it can be noticed that they appear at the intersections between the atomic debonding path and the Wallner lines. These local perturbations from the Wallner lines (underlying shear waves) generate locally more important SSMs whose amplitude increases strongly and then decays rapidly until they sink in lower SSMs. With the profile plots (Fig. 5e) carried out along the black dashed lines presented in Fig. 5f–h, we can notice that the central SSMs are similar to the lower ones in terms of spacing (0.5 vs. 0.5 μm) and amplitude (100 vs. 100 nm). Spacing between Wallner lines is larger, about 5 μm (see also Fig. 1d, e).

What can be the source of these SSMs that involve a strong velocity dependence? In our experiments, the silicon is of high purity (99.9999%), and no inclusions or voids have been observed on the fracture surfaces, so the SSMs are not a consequence of crack front–material defect interaction. They are not likely to initiate from the Rayleigh waves either, given the well distinguished spatial frequencies between the SSMs and the Wallner lines (see Fig. 5e). Moreover, the SSMs cannot be interpreted as multiple Wallner lines^25^, since the latter are usually observed when the crack initiates from a defect inside the solid, or in a case where external ultrasonic sources are present, both irrelevant to our fracture experiments. Another key argument to eliminate the material defects and Rayleigh waves is that they utterly fail to explain the velocity dependence of the SSM onset as well as the onset height in thickness. At this stage, we propose that the SSMs are intrinsic fracture products related to high crack velocity in (110) [110] cleavage. However, this kind of secondary surface marks have never been observed in tensile fracture tests, even for a crack velocity higher than 3000 m/s^8^. Hence, the SSM nucleation is likely related to the local crack velocity distribution under bending load.

### Front wave mechanism

**Fig. 6:**
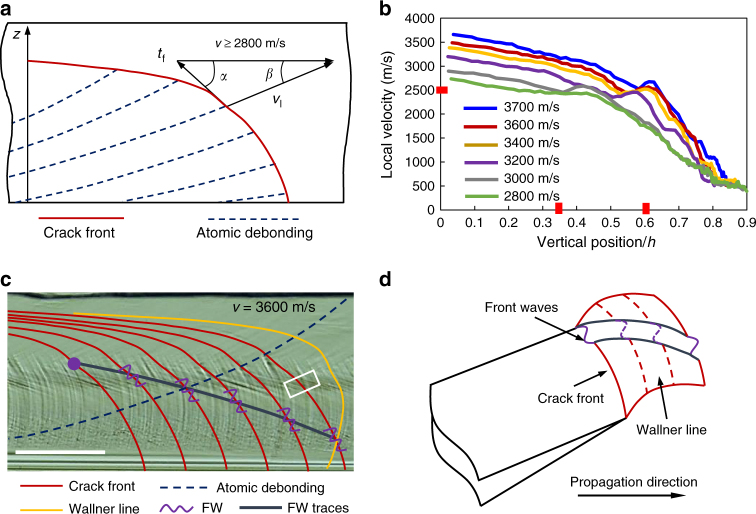
Analysis of the front wave origin and generation process. **a** Relationship between the local velocity *v*_l_ and the macroscopic propagation velocity *v*, *t*_f_ is the local tangent of the crack front. **b** Local velocity profiles with respect to different propagation velocities. **c** Real scale schema on the FW generation and propagation, the trace is compared with the experimentally observed SSM. **d** Superposition of the FW traces on the surface undulation (Wallner lines) in the framed region in **c**. Scale bar: 100 μm

The local crack velocity is correlated with the crack front shape. Therefore, the curvature jump, which appears jointly with the SSMs (see Fig. 3b), is certainly the manifestation of a local velocity irregularity. Considering the periodic wavy nature of the SSMs as well as their correlation with the presumed velocity irregularity, FW assumption is proposed. Note that FWs are physically localized pulses of in-plane velocity fluctuations, as numerically predicted in ref. ^26^. In order to highlight the expected irregularity, the local velocity along the crack front is addressed thanks to the following equation, according to Fig. 6a:

1 $$v_{\rm l} = \frac{{{\rm sin}(\alpha )}}{{{\rm sin}(\alpha + \beta )}}.v$$

Since the curvature jump impacts *α*, it will also modify *v*_l_. Six cases are now considered for the local velocity plotting, with the global steady-state velocities all higher than 2800 m/s. The results are shown in Fig. 6b. The first promising observation is that each considered local velocity profile involves a velocity fluctuation. This evidence constitutes a perfect rationale for the FW assumption in terms of the underlying driving force. More interestingly, it is found that all the fluctuations occur almost at the same local velocity, which is about 2500 m/s (see Fig. 6b). This allows us to explain why the SSM onset height rises when the global crack velocity increases, as can be noticed in Fig. 6b as well as Supplementary Fig. 2a. It is important to note that the shear waves that generate Wallner lines in a fracture process mainly have two sources, one regards the material flaws which induce an antiplanar shear component, and the other involves an ultrasonic transducer^34^. The in-plane velocity fluctuations therefore would not give rise to Wallner lines, given that mode I loading is maintained and no shear component will be derived. However, the FWs, though emanating from in-plane perturbations, involve an out-of-plane motion due to their nonlinear character^28, 35^, and thus produce surface marks, i.e., the so-called FW traces. The FW traces would be locally accompanied by mode III shear due to the out-of-plane components, but not necessarily by the Wallner lines. If Wallner lines were generated, the surface undulations would then be larger and larger in the lower part of the fracture surface because of the continuously generated Wallner lines. This hypothesis cannot be verified since the SSMs involve quite constant amplitudes, as shown in Fig. 5e, h. In order to further verify the FW assumption, the FW traces are produced and compared to the observed SSMs in the steady-state region of the rapid crack (3600 m/s). The production incorporates the real crack front shape, the crack velocity, the FW propagation velocity, i.e., the Rayleigh wave speed *C*_R_ as well as the corresponding curvature jump onset height, i.e., 0.57*h*. The comparison is addressed in Fig. 6c, a very good agreement can be observed. Another comparison is carried out for a crack velocity of 2800 m/s, the produced FW trace is once again coherent with the observed SSM, see Supplementary Fig. 3. As the FW propagates along the crack front, the FW traces will encounter the Wallner lines so that a superimposition of the two surface marks takes place. A schematic is drawn in Fig. 6d, which correlates well with the observation in Fig. 5g.

It has been highlighted in Fig. 6b that the local velocity fluctuation consistently emerges when the local velocity reaches about 2500 m/s. This characteristic allows excluding any extrinsic source for the FW nucleation and implies an intrinsic driving force. Here, we propose that the local velocity fluctuation is generated by the sudden increase in the dynamic toughness at a certain velocity, for instance 2600 m/s in the (110) [110] cleavage as reported in ref. ^8^. In the real fracture scenario, an accommodation will be established between the crack front shape (*α*), the local crack velocity (*v*_l_), and the static energy release rate (*G*_s_). Note that *v*_l_ forms *α*, *α* determines *G*_s_ under a given loading, while *G*_s_ feeds *v*_l_, the three interact in a closed circle as illustrated in Supplementary Fig. 4. In our case, due to the presence of the sudden increase in dynamic toughness, the local crack velocity oscillates, and the crack front simultaneously alters to follow the oscillation of the local velocity. The change in the crack front then affects the *G*_s_ distribution, which in turn adjusts the local velocity. These interactions proceed and terminate until the local velocity evolution along the crack front; the crack front shape and the *G*_s_ distribution converge to a mutual accommodation. Through the interactive process, the sudden increase in dynamic toughness intrinsically results in a velocity fluctuation and a curvature jump along the crack front. This in-plane velocity fluctuation then generates FWs which propagate along the crack front and produce the SSMs on the fracture surface.

## Discussion

The FW nucleation has been interpreted in light of the sudden increase in dynamic toughness in the (110) [110] cleavage at 2600 m/s^8^. Nevertheless, at the SSM onset site in our case, the local crack direction does not exactly head for the [110] direction, see Fig. 6a. Herein, the authors suppose that such toughness increase exists in other directions between [110] and [111] on the (110) plane at a velocity around 2500 m/s.

The FWs represent a mode of dissipation^28^. In the absence of external energy source, these waves can only propagate with the strain energy that flows into the crack front. However, in the fracture process under bending, the energy flux in the upper part is low and can barely drive the crack growth above the FW nucleation site, so little or no extra energy would be available for the FW propagation toward the upper part of the crack front. This explains why the SSMs appear only below the FW nucleation site where the energy flux is much higher.

According to our explanation, the FW would emanate for any crack velocity higher than 2500 m/s, while the FW traces have been experimentally observed only when the crack velocity exceeds 2800 m/s. It can be seen in Fig. 6b that the fluctuation becomes smaller as the steady-state velocity decreases and is barely noticeable at 2800 m/s. Hence, one may assume that the effect of velocity fluctuation is negligible when the steady-state velocity varies between 2500 and 2800 m/s. In this circumstance, the FWs barely initiate and propagate, which explain why no noticeable traces are left on the fracture surface.

In summary, it is found that (110) [110] cleavage of single crystalline silicon is highly stable and free of plane deflection under pure bending load, even at an extremely high velocity of 3700 m/s. The crack front shape has been reconstructed from the Wallner lines and revealed to have a strong dependence on the crack velocity. Due to the toughness increase at 2500 m/s, a local velocity fluctuation arises in high-speed cracks (above 2800 m/s) and manifests as a curvature jump along the crack front. Consequently, FWs emanate following the velocity fluctuation and generate secondary marks on the fracture surface, even in the absence of material asperity. The authors assume that such SSMs—or FW traces—could be observed in other crystalline materials with similar dynamic toughness evolution as in silicon.

## Methods

### Sample preparation

The specimens were obtained from solar grade silicon ingot with diamond wire sawing which results in a thickness around 200 μm and a square surface of 50 × 50 mm^2^. Chemical etching was carried out to eliminate a small layer of the damaged surface, while the saw marks were intentionally kept in order to generate fracture surface marks. A pre-crack was introduced for each specimen with a Vickers indent, employing controlled forces to ensure different defect sizes.

### Fracture tests

The fracture tests were carried out using a 4-line bending set up. 4-line bending was selected instead of 3-line bending to have a uniform bending stress between the central rollers, and also to get rid of the contact perturbations in the fracture region. The inner and outer spans are 21 mm and 40 mm, respectively. The specimens were bent to fracture under a quasi-static loading (10^−6^/s) with a LLOYD-Ametek LFPLUS electro-mechanical machine. All the tests were conducted under the same experimental conditions.

A Phantom V710 high-speed camera was set up in order to record the crack process thanks to a 45° tilted mirror between the two support rollers (refer to ref. ^22^ for more information about the experimental setup). The image resolution was fixed to 512 × 64 pixels, in combination with an acquisition frequency equal to 180,000 Hz. The camera recording was controlled by a trigger allowing saving the last 2 s of the images.

### Fracture surface evaluation

After the fracture tests, each crack surface was observed with a digital microscope (Keyence VHX-2000) to analyze the fracture surface morphology. High magnification fractographies were obtained with a scanning electron microscope (Oxford Instruments) to highlight tiny fracture surface marks. The nano-metric 3D information of these marks was obtained thanks to the atomic force microscope AFM PSIA XE-15, with an in plane resolution of 50 nm and a 1 nm resolution in height. The AFM topographies were analyzed with the WSxM software^36^.

### Data availability

Data supporting the findings of this study are available from the corresponding authors on reasonable request.

## Electronic supplementary material

Supplementary Information(PDF 358 kb)
