# Supplementary material for: Velocity correlated crack front and surface marks in single crystalline silicon
Source: Nat Commun. 2018 Apr 3;9:1298. doi: 10.1038/s41467-018-03642-w (PMC5880814; doi:10.1038/s41467-018-03642-w)
Supplement: Supplementary file 1 — Supplementary Information(PDF 358 kb) [file 41467_2018_3642_MOESM1_ESM.pdf]

Velocity correlated crack front and surface marks in single crystalline silicon

Zhao *et al.*

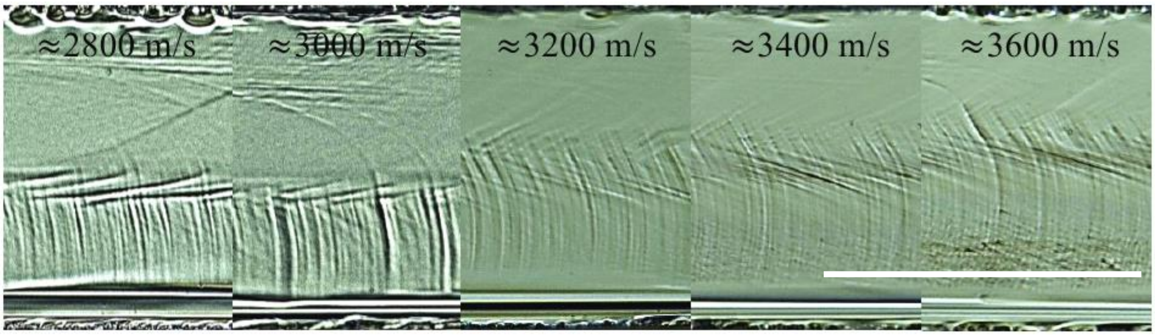

Supplementary Figure 1: Morphology of the special surface markings during the steady-state phase at various propagation velocities. Scale bar: 200 µm

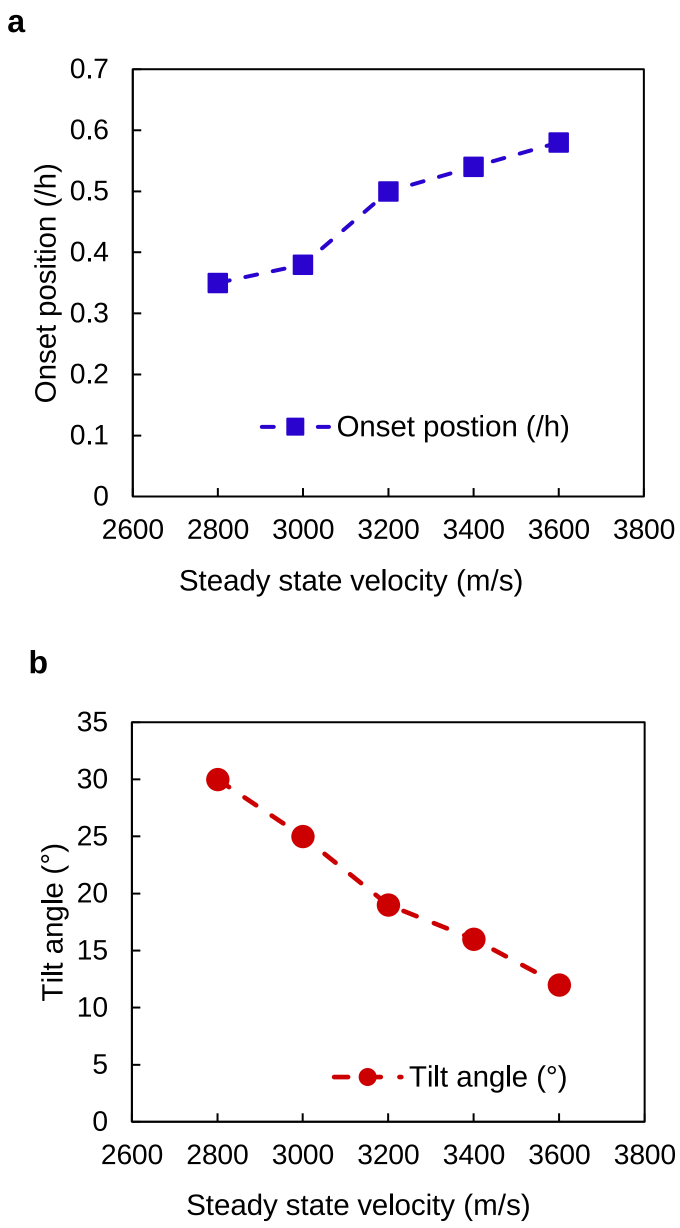

Supplementary Figure 2: Velocity dependent characteristics of the special surface marks. **a** onset height normalized by the specimen thickness, **b** tilt angle of the marks.

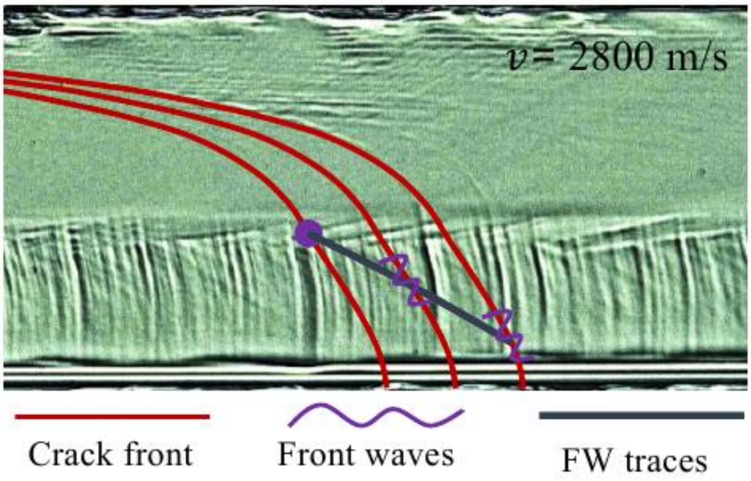

Supplementary Figure 3: Real scale scheme on the front wave generation and propagation, the trace is compared with experimentally observed special surface markings for a steady state velocity of 2800 m/s.

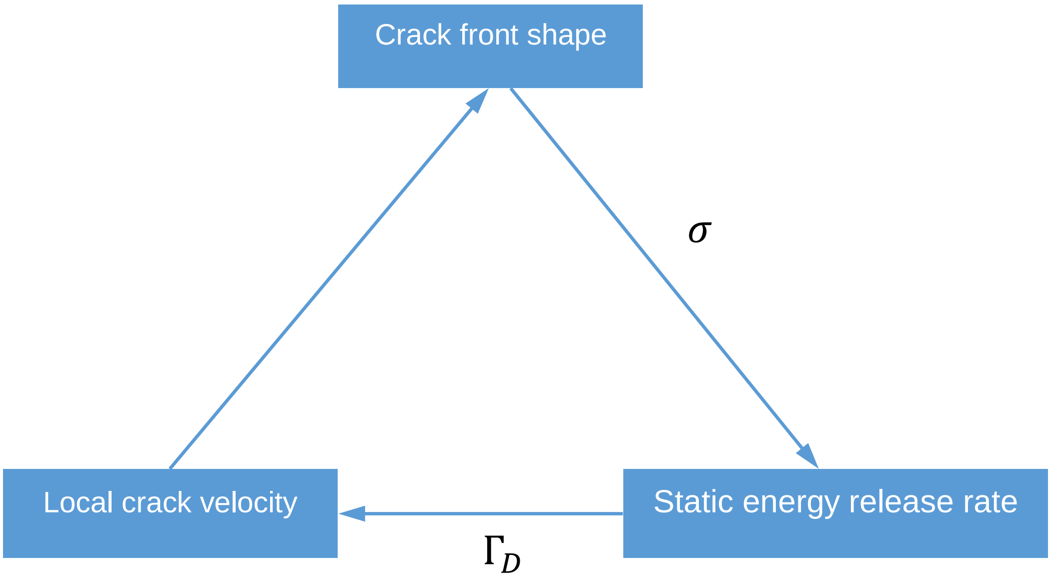

Supplementary Figure 4: Accommodation between the crack front shape, the local crack velocity and the static energy release rate under a given loading: local crack velocity forms the crack front shape, the crack front shape determines the static energy release rate, and static energy release rate feeds the local crack velocity.
